# Pelvic Floor Pressures Differ Based on Location in the Pelvis and Body Position: A Cadaver Mode

**DOI:** 10.3390/bioengineering10030329

**Published:** 2023-03-06

**Authors:** Tova Ablove, Alexandra DeRosa, Steven Lewis, Katelyn Benson, Frank Mendel, Scott Doyle

**Affiliations:** Jacobs School of Medicine and Biomedical Sciences, University at Buffalo, Buffalo, NY 14203-1121, USA

**Keywords:** pelvic organ prolapse, pelvic floor structure, pressure analysis, pelvic floor modeling, biomechanics

## Abstract

Background: The pelvic floor is a bowl-shaped complex of multiple muscles and fascia, which functions to support the pelvic organs, and it aids in controlling continence. In pelvic floor disease, this complex becomes weakened or damaged leading to urinary, fecal incontinence, and pelvic organ prolapse. It is unclear whether the position of the body impacts the forces on the pelvic floor. Purpose: The primary objective of this work is to measure force applied to the pelvic floor of a cadaver in sitting, standing, supine, and control positions. The secondary objective is to map the forces across the pelvic floor. Methods: An un-embalmed female cadaver without pelvic floor dysfunction was prepared for pelvic floor pressure measurement using a pressure sensory array placed on top of the pelvic floor, and urodynamic catheters were placed in the hollow of the sacrum, the retropubic space, and at the vaginal apex. Pressure measurements were recorded with the cadaver in the supine position, sitting cushioned without external pelvic floor support, and standing. Pressure array data were analyzed along with imaging of the cadaver. Together, these data were mapped into a three-dimensional reconstruction of the pressure points in pelvic floor and corresponding pelvic organs. Results: pressures were higher at the symphysis than in the hollow of the sacrum in the standing position. Pressure array measurements were lowest in the standing position and highest in the sitting position. Three-dimensional reconstruction confirmed the location and accuracy of our measurements. Conclusions: The findings of increased pressures behind the symphysis are in line with the higher incidence of anterior compartment prolapse. Our findings support our hypothesis that the natural shape and orientation of the pelvis in the standing position shields the pelvic floor from downward forces of the viscera.

## 1. Introduction

The pelvic floor is a bowl-shaped group of muscles and connective tissue located in the pelvis. It is made up of the levator ani muscle complex, coccygeus muscle, and endopelvic connective tissue [1], coalescing into a diaphragm with a maximum thickness of 3 mm that closes the bottom of the pelvis [2]. The perforations (i.e., from the vagina, urethra, and anus) in the pelvic floor created by these organs inherently weaken the structure as a whole. This complex supports the pelvic organs (bladder, vagina, and rectum) while aiding in controlling continence of both urine and stool [3]. Moreover, expansion and contraction of those organs during urination, defecation, and birthing further stress the structure.

Pelvic floor dysfunction (PFD) occurs when the muscles or connective tissue of the pelvic floor weaken. According to the American Urogynecologic Society, the most common types of PFD are urinary incontinence, pelvic organ prolapse, and fecal incontinence [4]. PFD is more common among older women [3], as well as those with risk factors, including obesity, chronic constipation, chronic cough, childbirth, and prior hysterectomy [5]. PFD is also common in women with high physical activity, such as athletes [6]. It is a very common condition with an overall prevalence of 23.7% [3].

PFD can have a profound impact on quality of life. Patients report social isolation, relationship problems, and sexual dysfunction [7,8] as some of the quality of life issues faced with PFD. Although there are many preventative and corrective therapies including pelvic floor muscle training, weight loss, controlling constipation, and pessaries, the majority of women will eventually opt for surgery. The lifetime risk of surgery for PFD is 20% [2]. In addition, according to a report in 2010, PFD costs over $500 million a year on the healthcare industry for surgeries [9], demonstrating the profound societal impact.

The pelvic floor muscles are thin, and the associated connective tissue is weak as compared to abdominal connective tissue or muscle tendons [1]. The inherent anatomy of the pelvic floor muscles dictate that these skeletal muscles would have to be in constant contraction in order to support the weight of the abdomen and pelvic organs. This tissue does not appear strong enough to withstand large forces over long periods of time, and it does not have the energy available for sustainability. Yet, while PFD is a common condition, the majority of women do not develop dysfunction, despite exhibiting the risk factors listed above (childbirth, gravity, long-term physically strenuous activity) [2,3]. Therefore, the risk factors are likely associative and not causal or deterministic.

We believe that anatomical forces incident on the pelvic floor play a role in the risk of developing PFD. To our knowledge, a direct study of the distribution of these anatomical forces on the pelvic floor has not been done.

In this study, we focus on exploring the nature of the anatomical forces that play across the pelvic floor as a result of different body positions. Our hypothesis is that different body positions will demonstrate differing visceral force loads on the pelvic floor, with the lowest visceral pressures displayed in the standing position. Further, we hypothesize that visceral load will be greater closer to the pubic symphisis, especially in postures reducing the lordotic lumbar curve.

## 2. Methods

This study has been approved by the Institutional Review Board at the University at Buffalo.

### 2.1. Cadaver Preparation and Pressure Sensor Placement

A fresh (un-embalmed) 103 lb female cadaver was acquired from the University at Buffalo, Department of Pathology and Anatomical Sciences Anatomical Gift Program. The cadaver was donated by a woman who was 92 years of age at the time of death. No signs of pelvic floor dysfunction were evident upon examination. A low transverse abdominal incision was made to expose the junction between small and large bowels. After the distal ileum was tied off, contrast was injected into the small bowel. A 16 French Foley catheter was inserted into the bladder. Remaining retroperitoneal, blunt dissection was used to access the pelvic floor on the right side without disrupting midline structures. A 3 × 8 cm array of pressure sensors mounted in cloth-like material (Tactarry Model 5334, Pressure Profile Systems Inc.) was inserted directly on top of the pelvic floor [10]. To reduce force dissipation through the array, a lead backing plate 1/64″ thick was inserted on the bone side of the array. T-DOC urodynamic catheters were placed in the hollow of the sacrum, in the retropubic space (symphysis), and transvaginally at the vaginal apex. The urodynamic catheter pressure data were recorded and analyzed using the Laborie Goby Portable Urodynamic System (Laborie UDS) [11].

Measurements were taken transvaginally for pressures at the vaginal apex because this is a pressure commonly measured during standard urodynamic testing in women, and there are normal ranges published in the literature that could be compared to our findings as a control. The pressure array was tared and the abdominal incision closed. Lifting straps attached to a hydraulic lift were applied to the cadaver’s upper extremities. To simulate abdominal wall tone, a rubber belt was placed snugly around the trunk.

### 2.2. Cadaver Postures

Data were collected with the cadaver manipulated into four positions: standing, sitting, supine, and a control position where the cadaver was seated on a donut-shaped inflatable hemorrhoid cushion that, in theory, precluded external support of the pelvic floor when seated. For the first three positions we performed urodynamic pressure measurements (Laborie UDS), pressure array readings, and image capture as described below. For the donut-seated control position, we limited our capture to urodynamic pressure and pressure array readings to compare with published in vivo data (i.e., vaginal apex sensor in the sitting position on donut shaped hemorrhoid cushion).

### 2.3. Force Measurements

The cadaver was first placed in the supine position with the pressure array in place. Pressure measurements from the pressure array and the Laborie UDS (sacrum, symphysis, vaginal apex) were recorded electronically for approximately one minute. Via a hydraulic lift, the cadaver was then lifted into the sitting position where pressure measurements were again recorded for approximately one minute. Next, the cadaver was moved onto the hemorrhoid cushion, and measurements were recorded. Finally, the cadaver was lifted into the standing position (feet on the floor), and the pressure measurements were recorded.

For reproduceability, this series was repeated a total of ten times. During cadaver manipulation, an external video camera was used to record movements and was synchronized to the pressure array; this ensured that the sensor readings were taken when the cadaver was at rest.

**Figure 1:**
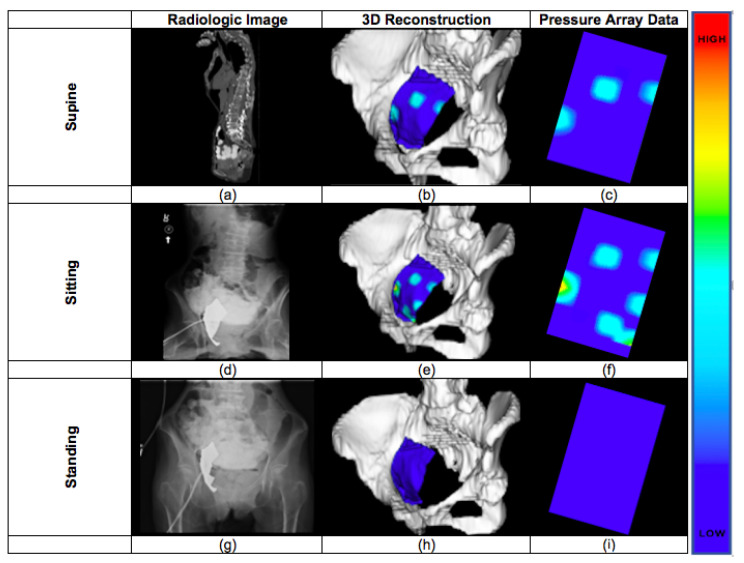
Examples of the body position radiology data with three-dimensional reconstructions of the bones of the pelvis with the pressure array data. (**a**,**d**,**g**) Radiological images of the cadaver with the pressure sensor implanted in supine, sitting and standing positions. (**b**,**e**,**h**) 3D reconstructions of the lumbar and pelvic bones with the pressure sensor mapping along the pelvic floor. (**c**,**f**,**i**) Images of the pressure array data taken without 3D reconstruction. The higher the pressure the more red the color, while the lower the pressure the more blue the color on the map.

Pressure array data were analyzed via Chameleon Tactile Visualization and Recording Software [12]. The total pressure in each position for each experiment was recorded in grams. A visualization of the pressures exerted on the array in each position for each experiment was saved for texture mapping in the three-dimensional reconstruction step Figure 1.

### 2.4. Statistical Analysis

Sacral and symphysis UDS pressures were compared via *t*-test. The effect of cadaver position on total array pressures and UDS vaginal apex pressures were compared using ANOVA, and post hoc tests were conducted using TukeyHSD. Analyses were conducted using the R statistical programming language [13].

### 2.5. Image Acquisition

Following the force measurement experiments, the cadaver was taken to Buffalo General Hospital for imaging. The Foley catheter, pressure array, and urodynamic catheters remained in place. A CT scan of the supine cadaver was first taken. A standing CT was not available. Therefore, multiple X-ray images were taken. The cadaver was first placed in a seated position where anterior-posterior, right lateral, left lateral, and oblique images were taken. The cadaver was then placed in the standing position (via the hydraulic lift), and the same series of images were again taken. The CT and X-ray images were saved on CDs for analysis.

### 2.6. Image Analysis and Three-Dimensional Reconstruction

**Figure 2:**
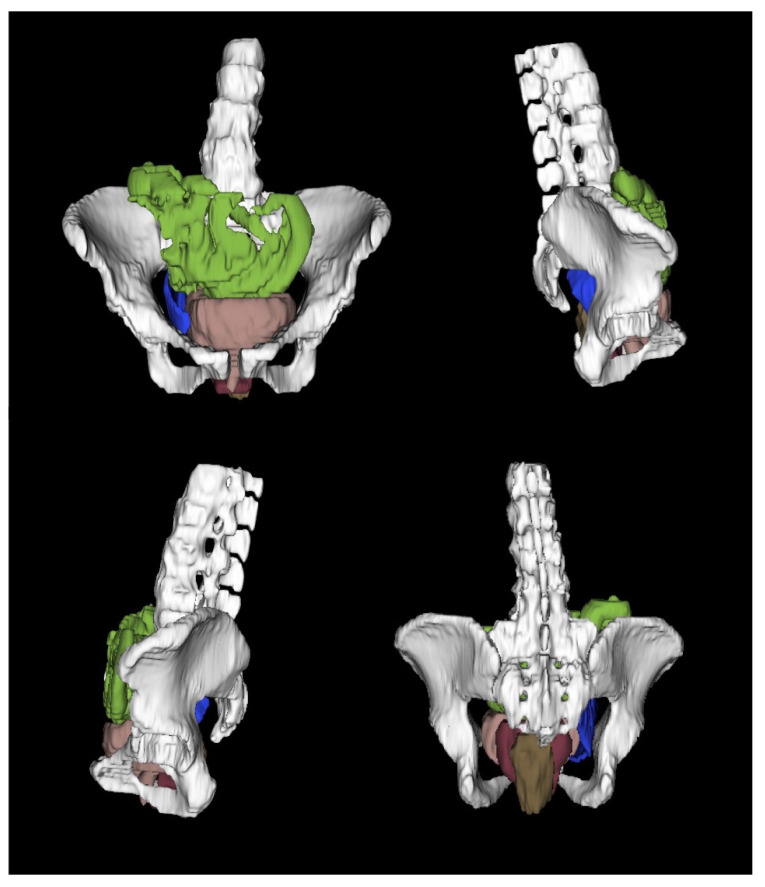
3D models of the pelvic floor with abdominal structures.

The supine CT images were uploaded into 3D Slicer 4.7 for analysis [14,15]. With use of the segment editor and editor modules, a three-dimensional model of the pelvic floor was constructed (Figure 2).

The lumbar spine with sacrum, pelvic bones, bladder, vagina, rectum, pressure array, and bowel were segmented. Each segment was loaded into a scene to create a three-dimensional model of the cadaver in the supine position. The X-ray images were utilized to model the cadaver’s pelvic floor in the sitting and standing positions. Each segment was aligned to the X-ray images via the transform module. A linear transform was created for each segment in each position to correctly orient the model with the X-ray images. The left column of Figure 1 shows the original radiologic data sets.

The images from the pressure array collected via Chameleon Tactile Visualization were utilized to create a texture in the MeshLab 3D modeling software [16]. Examples of this texture are shown in the right column of Figure 1, where blue colors correspond to low pressures, and red corresponds to high. The texture was then applied to the pressure array model in 3D Slicer for the various cadaver positions (the middle column of Figure 1). The tactile images of the array allowed for the mapping of pressure points within the context on the pelvic floor and the corresponding pelvic organs.

## 3. Results

### 3.1. Force Measurements (UDS and Pressure Array)

**Figure 3:**
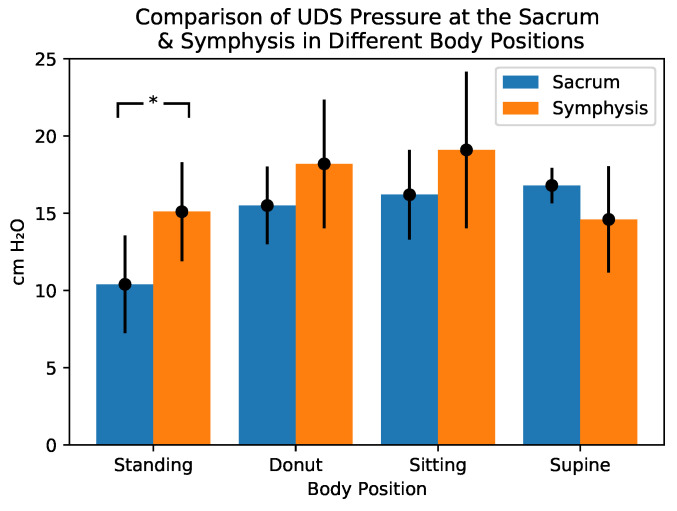
Comparison of the different pressures exerted on the pelvic floor in the different positions in the anterior vs. the posterior compartments (anterior being the symphisis region while posterior being the sacral region). Asterisks indicate statistically significant values.

**Figure 4:**
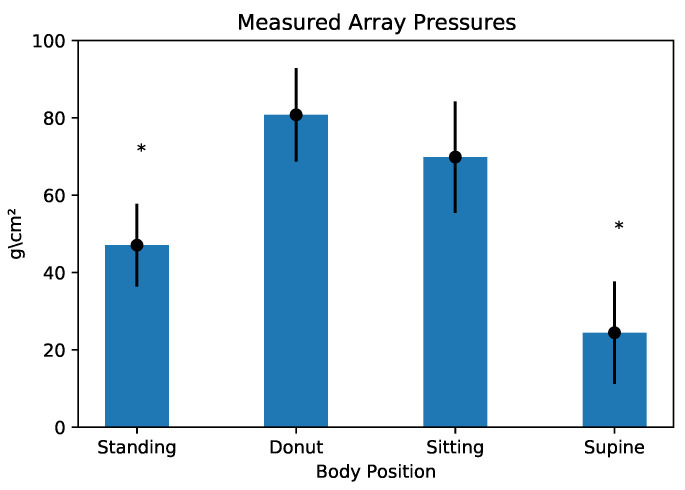
Overall pressures measured via the pressure array. Asterisks indicate statistically significant values.

**Figure 5:**
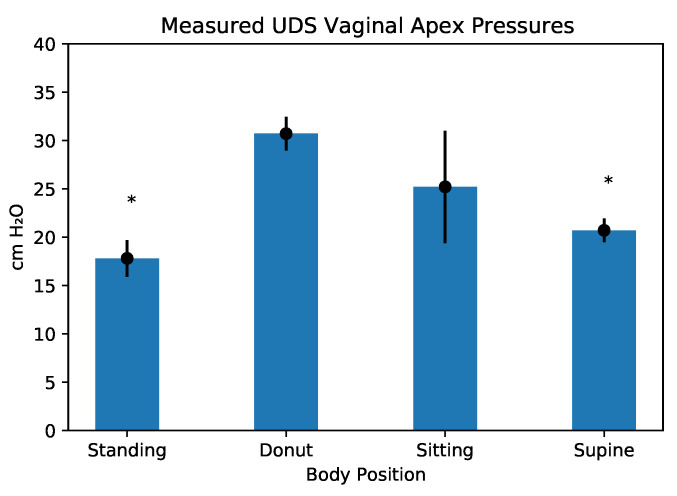
Pressures measured at the vaginal apex in the different positions. Asterisks indicate statistically significant values.

The results of the UDS pressure catheter measurements are shown in the plots in Figure 3, Figure 4 and Figure 5. The pressures recorded by the T-DOC catheters are in cm H${}_{2}$O whereas those recorded by the array are in gm/cm${}^{2}$ (Figure 3). For the pressure array, values are reported as mean (gm/cm${}^{2}$), standard deviation values over the rectangular array area. In each plot, the horizontal line indicates the median recorded value.

#### 3.1.1. Symphysis vs. Sacrum Comparisons

Pressures at the sacrum were significantly lower than those at the symphysis in the standing position (p=0.0037). There were no significant differences in pressures between sacrum and symphysis in the supine (p=0.08112), sitting (p=0.1335), or donut positions (p=0.0956) (Figure 3).

#### 3.1.2. Array Comparisons

Pressures were lowest and statistically different in the standing position (24.5 gm/cm${}^{2}$, SD 13.2) when compared to the supine (47.1 gm/cm${}^{2}$, SD 10.7), sitting (80.8 gm/cm${}^{2}$, SD 12.1), and donut positions (69.9 gm/cm${}^{2}$, SD 14.4). Pressure in the supine position was found to be lower and statistically different from those in the sitting and donut positions (Figure 1 and Figure 4).

#### 3.1.3. Vaginal Apex Comparisons

Pressures in the standing and supine positions were similar to each other (p=0.21), but they were lower and statistically different from those in the sitting (p<0.01) and donut positions (p<0.01). Pressures in the sitting and donut position were statistically different from each other (p<0.01) (Figure 5).

### 3.2. Image Acquisition Results

The images acquired via X-ray and CT are shown in Figure 1. The CT image was acquired in the supine position (top row), whereas X-ray images were acquired in the sitting and standing positions. In all of the imaging modalities, the pressure array was clearly identifiable; there was no observable movement of the array relative to other structures in the pelvic throughout manipulation of the cadaver on the hydraulic lift. Although the pressure array was fixed with a lead backing, the presence of a metal artifact did not preclude our ability to model the pelvis and surrounding soft tissues.

### 3.3. Three-Dimensional Modelling

The images of the pressure array are shown in Figure 1. Red colors on the array indicate increasing pressures, and blue represents zero pressure. Pressures were not evenly distributed across the array, but instead were specific to geographic locations. Pressure increases were observed where viscera contacted the array.

## 4. Discussion

The purpose of this study was to directly measure pressure experienced in the pelvic cavity in different postures and anatomic locations. Placing a UDS sensor at the vaginal apex is a common means of measuring abdominal pressures in vivo. To further simulate live urodynamic testing, we placed the cadaver on a hemorrhoid donut removing external support, as would be standard when sitting on a commode in the urodynamic suite. In addition, we placed UDS sensors in locations that cannot be measured in vivo. These were placed behind the pubic symphysis (in the space of Retzius) near the anterior floor of the pelvis, as well as in the hollow of the sacrum (near the roof of the pelvis). We also measured total force on the pelvic floor using the Pressure Profile Systems array positioned directly on top of the levator ani muscles, which also cannot be done in vivo. We hypothesized UDS pressures would be highest anteriorly, and array pressures would be lowest in the standing position

UDS pressures were indeed higher anteriorly, and array pressures were lowest in the standing position. UDS measured sitting and supine measurements, which were consistent with normative in vivo findings, supporting the validity of the model [17,18]. Reported intraabdominal pressure readings obtained similarly through a Foley catheter were 6.5 mm Hg with a range of 0.2–16.2 mm Hg [19], and in various body positions at 9.5 cm H_2_O for supine, 11.5 cm H_2_O at 30 degrees, and 14 cm H_2_O at 45 degrees. In addition, intravaginal measurement of intraabdominal pressures have been reported at 27.5 cm H_2_O [20] and in the supine position at 1.5 cm H_2_O [21]. Finally, for the morbidly obese, intraabdominal pressures have been reported at 12.0 cm H${}_{2}$O, which was compared to the control of 0–1.2 cm H${}_{2}$O.

The way the pressures are generated at the vaginal apex are very different from those in the abdominal cavity, and, for this reason, we did not directly compare them. At the vaginal apex, the UDS sensor is sandwiched between the anterior and posterior vaginal walls. The distal vagina is normally oriented along the horizontal plane and is compressed by downward internal forces. The compression of the distal vagina is likely a protective mechanism.

Noblett et al. [20] reported higher pressures in the sitting position compared to the supine using an intravaginal pressure catheter. Our study also noted higher pressures in the sitting compared to the supine position at the vaginal apex. Interestingly, pressures were higher over the levators and behind the symphysis in the sitting position. At the hollow of the sacrum, pressures in the sitting and supine position were similar and both higher than standing. Shaw et al. [21] measured abdominal pressures using an intravaginal sensor in studies designed to evaluate how exercise affected intra-abdominal pressure. They noted increased abdominal pressures in obese patients and did not report measurements at rest. Several studies measured visceral pressures as a surrogate for abdominal pressures, and they found that obesity increased abdominal pressure [19,22,23]. This effect of increased weight in the viscera, seen in truncal obesity, supports the idea that the small bowel plays a role in pressure applied to the pelvic floor. There are no studies in live subjects or cadavers measuring abdominal pressures at various locations within the pelvis, nor are there any studies showing these pressures as a result of differing body positions.

This research helps explain the higher incidence of anterior compartment prolapse in women with pelvic floor disease. Pressures were higher at the symphysis than in the hollow of the sacrum in the standing position, supporting our hypothesis. Values were lowest in standing position (sacrum has maximum shielding) and highest in sitting position (sacrum has diminished shielding), potentially due to loss of lumbar lordosis when sitting. Pressures measured in the donut position were between the sitting and standing positions.

### Imaging and Three-Dimensional Modeling Are Consistent with Pressure Readings

Our experiments were performed on a cadaver, which initially raised concerns about the relevance of our findings for in vivo conditions. In an effort to address this concern, we measured pressures at the vaginal apex using the Laborie Gomi UDS machine, as we would during an in vivo UDS procedure. We positioned the cadaver in the sitting position on a donut to simulate sitting on the UDS chair. Our measurements at the vaginal apex were consistent with in vivo results. We repeated the experiment 10× to confirm its reproducibility. We also had concerns regarding the reliability of our array and its position in the pelvis. We confirmed the position of the array using CT scan, X-ray, and three-dimensional modeling. We found that the array was precisely positioned over the levator ani muscles abutting the midline structures. The small bowel moved with changes in position and generated pressures measured by the array, which was confirmed with our imaging.

The three-dimensional modeling of the pelvic region was performed in 3D Slicer to verify the positions of internal organs, as well as the pressure array (Figure 2). The use of radiology, and specifically of magnetic resonance imaging (MRI), has been well established in the assessment of pelvic floor anatomy, identification of dysfunction, and assessment of surgical outcomes.

The use of non-contrast-enhanced cadaveric CT scanning for generating three-dimensional models presents a set of unique challenges. Lack of contrast between soft tissue organs decreases the fidelity of the structures of interest, and, while cadaveric contrast agents are being developed, they are not yet widely available or well studied. Thus, our segmentation and analysis is limited to tissues with clear boundaries (i.e., bones, bladder, intestines). Thin structures, such as the pelvic floor itself and microvessels, were difficult to visualize and model, and so were inferred from the surrounding anatomy. Additionally, without a standing CT apparatus, the assessment of the mesh locations in non-supine positions needed to be inferred from other imaging methods (X-ray). As far as we are aware, the positioning of the mesh was not significantly altered from the movement of the cadaver, but this should be confirmed by additional imaging.

## 5. Conclusions

Our findings of increased pressures behind the symphysis are in line with the known higher incidence of anterior compartment prolapse. In addition, the notion that the natural shape and orientation of the pelvis with standing helps to protect the pelvic floor is supported by our findings of lower pressures at the hollow of the sacrum in the standing position.

This study was conducted using a single sample body to generate a set of pilot readings. We were unable to calculate average and variation of pressures across different body morphologies; future work should expand the sample size to look at whether these pressure readings hold across different ages and pelvic floor states (dysfunction vs. normal).

Additionally, due to the nature of the study, we used a human gift cadaver to record pressures directly on the pelvic floor. Such an invasive methodology is not feasible to perform on living patients, and we do not know the extent to which our findings are directly translatable to living individuals. Our specimen was, likewise, from an elderly individual (aged 92 years) and, therefore, may not be translatable to the general condition.

The magnitude of our UDS pressure recordings are in line with previously reported findings, suggesting that the overall findings are at least reasonably comparable with the pressures one could expect from living subjects. More detailed and less invasive pressure recording approaches will be needed before these findings can be replicated on pre-mortem patients [19,20,21,22,23]. The potential applications of these results may demonstrate that lower lumbar lordosis treatment can alleviate pelvic floor dysfunction.

## Data Availability

Data are not available at this time.

## Abbreviations

The following abbreviations are used in this manuscript:

PFD	Pelvic Floor Dysfunction
UDS	Urodynamic System
ANOVA	Analysis of Variance

## References

[1] Herschorn S. (2004). Female Pelvic Floor Anatomy: The Pelvic Floor, Supporting Structures, and Pelvic Organs. Rev. Urol..

[2] Wu J.M., Vaughan C.P., Goode P.S., Redden D.T., Burgio K.L., Richter H.E., Markland A.D. (2014). Prevalence and Trends of Symptomatic Pelvic Floor Disorders in U.S. Women. Obstet. Gynecol..

[3] Nygaard I., Barber M.D., Burgio K.L., Kenton K., Meikle S., Schaffer J., Spino C., Whitehead W.E., Wu J., Brody D.J. (2008). Prevalence of symptomatic pelvic floor disorders in US women. JAMA.

[4] What Are PFDs?–About | Voices for PFD. https://www.voicesforpfd.org/about/what-are-pfds/.

[5] Good M.M., Solomon E.R. (2019). Pelvic Floor Disorders. Obstet. Gynecol. Clin. N. Am..

[6] Nygaard I.E., Shaw J.M. (2016). Physical activity and the pelvic floor. Am. J. Obstet. Gynecol..

[7] Mazi B., Kaddour O., Al-Badr A. (2019). Depression symptoms in women with pelvic floor dysfunction: A case-control study. Int. J. Womens Health.

[8] Verbeek M., Hayward L. (2019). Pelvic Floor Dysfunction And Its Effect On Quality Of Sexual Life. Sex. Med. Rev..

[9] Sung V.W., Washington B., Raker C.A. (2010). Costs of ambulatory care related to female pelvic floor disorders in the United States. Am. J. Obstet. Gynecol..

[10] Pressure Profile Systems Inc. Systems^®^|Tactile Pressure Mapping Systems. https://pressureprofile.com/.

[11] Laborie. https://www.laborie.com/.

[12] Pressure Profile Systems Inc. Chameleon Visualization Software. https://pressureprofile.com/sensor-systems/software.Date.

[13] R: The R Project for Statistical Computing. v4.1.3. https://www.r-project.org/.

[14] Fedorov A., Beichel R., Kalpathy-Cramer J., Finet J., Fillion-Robin J.C., Pujol S., Bauer C., Jennings D., Fennessy F., Sonka M. (2012). 3D Slicer as an Image Computing Platform for the Quantitative Imaging Network. Magn. Reson. Imaging.

[15] Kikinis R., Pieper S.D., Vosburgh K.G., Jolesz F.A. (2014). 3D Slicer: A Platform for Subject-Specific Image Analysis, Visualization, and Clinical Support. Intraoperative Imaging and Image-Guided Therapy.

[16] Cignoni P., Callieri M., Corsini M., Dellepiane M., Ganovelli F., Ranzuglia G. MeshLab: An Open-Source Mesh Processing Tool. Proceedings of the Eurographics Italian Chapter Conference.

[17] Nager C.W., Albo M.E., Fitzgerald M.P., McDermott S., Wruck L., Kraus S., Howden N., Norton P., Sirls L., Varner E. (2007). Reference urodynamic values for stress incontinent women. Neurourol. Urodyn..

[18] Schäfer W., Abrams P., Liao L., Mattiasson A., Pesce F., Spangberg A., Sterling A.M., Zinner N.R., van Kerrebroeck P. (2002). Good urodynamic practices: Uroflowmetry, filling cystometry, and pressure-flow studies. Neurourol. Urodyn..

[19] Sanchez N.C., Tenofsky P.L., Dort J.M., Shen L.Y., Helmer S.D., Smith R.S. (2001). What is normal intra-abdominal pressure?. Am. Surg..

[20] Noblett K.L., Jensen J.K., Ostergard D.R. (1997). The relationship of body mass index to intra-abdominal pressure as measured by multichannel cystometry. Int. Urogynecol. J. Pelvic Floor Dysfunct..

[21] Shaw J.M., Hamad N.M., Coleman T.J., Egger M.J., Hsu Y., Hitchcock R., Nygaard I.E. (2014). Intra-abdominal pressures during activity in women using an intra-vaginal pressure transducer. J. Sport. Sci..

[22] Lambert D.M., Marceau S., Forse R.A. (2005). Intra-abdominal pressure in the morbidly obese. Obes. Surg..

[23] Chionh J.J.L., Wei B.P.C., Martin J.A., Opdam H.I. (2006). Determining normal values for intra-abdominal pressure. ANZ J. Surg..

